# Case-based Clinical Reasoning Competitions: A Novel Method to Teach and Assess Clinical Reasoning Skills Among Gastroenterologists

**DOI:** 10.5152/tjg.2024.24024

**Published:** 2024-10-01

**Authors:** Anahita Sadeghi, Peyman Adibi, Fatih Oğuz Önder, Bülent Değertekin, Dilara Turan, Serhat Bor

**Affiliations:** 1 Tehran University of Medical Sciences Digestive Disease Research Institute, Tehran, Iran; 2 Isfahan University of Medical Sciences, Isfahan, Iran; 3 Acıbadem Mehmet Ali Aydinlar University, İstanbul, Türkiye; 4 Department of Gastroenterology, Ankara Bilkent City Hospital, Ankara, Türkiye; 5 Ege University School of Medicine, İzmir, Türkiye

Dear Editor,

Clinical reasoning is a complex and essential skill for medical doctors, including gastroenterologists, who often encounter challenging cases that demand real-time problem-solving. To teach and assess clinical reasoning skills, we conducted a case-based clinical reasoning competition during the 40th National Gastroenterology Week in Antalya, November 2023.

The competition consisted of 2 parts: a morning session with 4 stations presenting clinical scenarios that needed to be solved by the contestants, and an evening session with presentations of case scenarios prepared by the contestants based on a random diagnosis. The competition was scored by raters and the audience using a scoring system that emphasized high-value care, timely decision-making, prompt life-saving treatment plans, and avoiding unnecessary investigations. The feedback from the raters, the contestants, and the audience was positive and indicated that the competition was an engaging and stimulating learning event that enhanced clinical reasoning skills and motivation. We conclude that case-based clinical reasoning competitions are a valuable tool for teaching and assessing clinical reasoning skills among gastroenterologists, and we recommend incorporating them into medical education and practice.

Imagine you are a gastroenterologist who is faced with a patient with severe constipation who does not respond to any conventional treatment. How would you approach this case? What kind of reasoning skills would you need to solve this problem? This is the kind of challenge that participants of the Case-based clinical reasoning competitions had to deal with during the 40th National Gastroenterology Week in Antalya, November 2023.

Clinical reasoning is a complex process by which clinicians collect, process, and interpret patient information to develop an action plan that involves both conscious and unconscious cognitive activities.^1,2^ There are 2 types of reasoning: analytical reasoning and pattern recognition, also known as intuitive or heuristic reasoning.^2^ Each type of processing has its pros and cons, and these 2 approaches are not mutually exclusive—they often work together to reach a final diagnostic or therapeutic decision. Clinical reasoning is an essential skill for all medical doctors, regardless of their specialty. It is one of the core competencies that every physician should master, and medical educators should focus on teaching and evaluating this skill.^1,3^

Case-based clinical reasoning competitions may be an effective and engaging way to teach and assess clinical reasoning skills and simulate real-life clinical scenarios and problems that require clinical reasoning skills. We conducted a case-based clinical reasoning competition to create an engaging and stimulating learning event for the participants.

The first draft of the details about clinical scenarios, questions, scoring system, and the scorekeeping system was prepared by 5 faculty members. They were revised and edited in several online sessions. The following goals were considered in the scoring system: High-value care, timely decision-making, prompt life-saving treatment plans, and avoiding unnecessary investigations.

The competition format was a case-based competition with the target audience being young clinicians and GI fellows. The competition was divided into 2 parts.

First, the executive team announced the competition and persuaded GI fellows to participate. Among more than 30 applicants, 18 were randomly recruited, and 4 groups were formed. Each group included 4-5 members, consisting of GI fellows and GI surgeon fellows from Türkiye, Asia, and Europe. The competition structure was sent to all participants before the competition. A briefing session was held on the day of the competition. The goal and the instructions of the competition were explained to the contestants, and their questions were answered. The first part of the competition consisted of 4 stations, each presenting a clinical scenario with problems that needed to be solved by the contestants. (Figure 1)

After hearing the bell ring, each group moved clockwise to the next station until they finished all 4 stations. The raters scored the teams at each station based on a checklist and a global rating form. The teams had 10 minutes to answer and solve the scenarios at each station.

In the evening, a second briefing session was held, and clinical reasoning and high-value care were briefly explained. The goal and the instructions of the competition were also clarified to the contestants, and their questions were addressed. Each team drew 1 paper by lottery from a bottle that contained 4 papers, each with a different diagnosis. To randomize the selection process, we first assigned numbers to the groups by chance. The number determined the order of choosing their option. Each team prepared their case scenario in 2 hours, which was presented in 10 minutes in front of the jury team and the congress audience. All presentations were scored regarding history taking (10 marks), physical examination (5 marks), diagnostic evaluation (10 marks), treatment (10 marks), and global rating (15 marks). The audience also rated them through the Congress App. The morning and evening sessions each contributed 50% to the competition’s total score.

After the competition, we had a semi-structured interview with all referees and also took feedback from the audience and contestants in small group sessions.

The referees pointed out that the teams worked together to solve the clinical problems and that they communicated effectively, shared their perspectives, and reached a consensus. The contestants said that the raters provided constructive feedback to the teams on their performance, highlighting their strengths and weaknesses. They also mentioned that the case-based clinical reasoning competitions were an opportunity to showcase their skills and knowledge and also enhance motivation, interest, and enjoyment of learning and teaching clinical reasoning (Figure 2).

We believe that case-based clinical reasoning competitions are a valuable tool to enhance clinical reasoning skills among gastroenterologists.

As organizers, we have learned how to design realistic cases and facilitate competitions, and we would like to increase the frequency and variety of the competitions while improving their quality and impact by introducing new formats and themes, organizing online or regional competitions, and developing standardized criteria and tools for evaluating participants’ performance.

We encourage medical educators and practitioners to adopt this method in their curricula and continuing education programs. We also invite researchers to conduct further studies on the effectiveness and impact of this method on clinical outcomes and patient care.

## Figures and Tables

**Figure 1.**
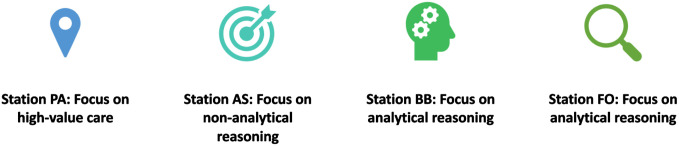
The goal of each station.

**Figure 2.**
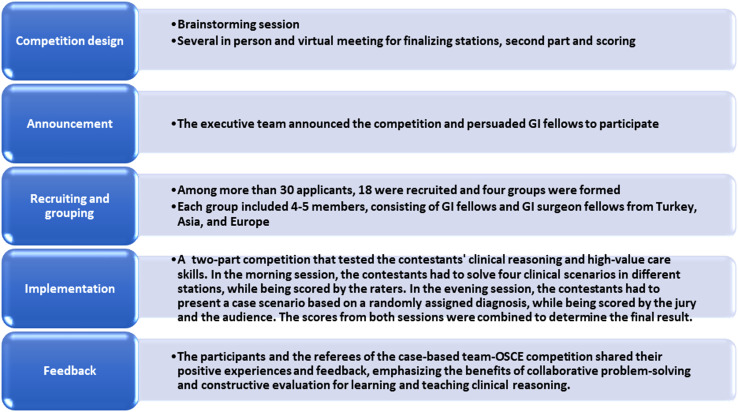
Summary of competition.
